# Progress of Ecological Restoration Research Based on Bibliometric Analysis

**DOI:** 10.3390/ijerph20010520

**Published:** 2022-12-28

**Authors:** Xi Wei, Wei Song, Ya Shao, Xiangwen Cai

**Affiliations:** 1 Key Laboratory of Land Surface Pattern and Simulation, Institute of Geographic Sciences and Natural Resources Research, Chinese Academy of Sciences, Beijing 100101, China; 2 School of Earth Sciences, Guilin University of Technology, Guilin 541000, China; 3 Hebei Collaborative Innovation Center for Urban-Rural Integration Development, Shijiazhuang 050061, China

**Keywords:** ecological restoration, research focus, research direction, Web of Science, Bibliometrix

## Abstract

With the deterioration of the global/regional ecological environment, ecological restoration plays an important role in sustainable development. However, due to the differences in research methods, objectives, and perspectives, the research results are highly diverse. This makes it necessary to sort the publications related to ecological restoration, clarify the research status, grasp the research hotspots, and predict the future research trends. Here, 23,755 articles from the core database of Web of Science were retrieved, and bibliometric analysis was carried out to understand the global ecological restoration research progress from 1990 to 2022 from a macro perspective, with the aim to determine the future development direction. The results are as follows. (1) From 1990 to 2022, the number of publications in the field of ecological restoration constantly increased, and the fluctuation of the average annual citations increased. The most important articles were published in high-ranking journals. (2) Ecological restoration covers a wide range of research areas, including biodiversity, ecosystem services, climate change, land use, and ecological restoration theories and technologies. The four main hotspots in this field are heavy metal removal, soil microbial biomass carbon and nitrogen concentrations, grassland ecological restoration, and evaluation framework and modeling of ecological restoration’s effects. Currently, studies focus on river basin remediation, heavy metal removal, and forest restoration. (3) Future ecological restoration research should strengthen the multi-object aspect and multi-scale ecological restoration research, improve the ecological restoration effect evaluation system, and incorporate social and economic issues. This study identified current research hotspots and predicted potential future research directions, providing a scientific reference for future studies in the field of ecological restoration.

## 1. Introduction

Since the industrial revolution, with the rapid development of the global economy, people’s material living standards have been improving constantly. However, this goes along with issues such as global warming, environmental pollution, and loss of biodiversity [1,2]. Natural ecosystems, such as forests, grasslands, and wetlands, are in massive decline globally [3]. Ecosystem structure and function are being destroyed on a large scale, the quality of the ecological environment is deteriorating [4], natural resource scarcity is becoming increasingly critical [5], and the food supply and service capacity of ecosystems are declining sharply, seriously threatening the living environment of human beings and the sustainable development of society and the economy [6]. In this context, the restoration of degraded ecosystems has attracted great attention around the world.

Numerous countries and regions have taken a range of measures to address issues such as ecosystem degradation. In the 1950s–1960s, Europe and North America pioneered a series of ecological projects to address the ecological crisis [7]. In 2010, the Conference of the Parties to the Convention on Biological Diversity (CBD), in its *Strategic Plan for Biodiversity 2011–2020*, specified the restoration of at least 15% of degraded ecosystems [8]. In a resolution in 2019, the United Nations General Assembly, led by the United Nations Environment Programme (UNEP) and the Food and Agriculture Organization of the United Nations (FAO), declared the United Nations Decade for Ecosystem Restoration (2021–2030) to address biodiversity loss, climate disruption, and increased pollution [9].

The term “ecological restoration” was proposed by Leopold in 1935 [10]. Ecological restoration focuses on the ecosystem as an object [11], aiming to repair damaged ecosystems and rebuild species composition and structure, with emphasis on the improvement and overall enhancement of ecosystems [12]. The goal is to establish a balanced ecosystem that meets the requirements of social and economic development through the reconstruction and restoration of the ecosystem, relying both on the inherent restoration ability of the ecosystem and on the intervention of science and technology [13]. Science and technology means mainly include physical, chemical, bioremediation, microbial, animal, and plant remediation methods. For example, Sun et al. [14] adopted the design method of horizontal subsurface-flow constructed wetlands to restore the ecological environment of the Yanxi River Basin, an important water resource conservation area in Beijing. Grant et al. [15] used smoldering technology to restore coal-tar-contaminated sites. Wu et al. [16] constructed a remediation system for cadmium-polluted soil using biochar, pro-growth bacteria, and super-accumulative plants. Ecological restoration can be classified into large-scale (e.g., on the Loess Plateau [17]), medium-scale (e.g., river channels [18], water bodies [19], forests [20], wetlands [21]), and small-scale ecological restoration (e.g., mines [22], landfills [23]). Based on the restoration object, scientists distinguish mountain [24], river [25], soil [26], and plant ecological restoration [27], among other types. There is also the view that ecological restoration needs to be enhanced through social channels [28]. As problems such as ecosystem degradation are caused by unsustainable human activities, as in the case of land degradation caused by slope planting, people who are highly dependent on these activities are urged to make some changes [29]. This will largely determine the success of ecological restoration and the healthy and sustainable development of the ecosystem if ecological restoration balances conflicting ecological and social interests. In addition, scientists are largely aware that ecological restoration also involves various challenges such as poverty, food security, and urbanization [30,31]. Ecological restoration must involve not only the restoration of destroyed ecosystems but also the protection and management of intact ecosystems [32,33]. The status quo and restoration effect of the ecosystem can be evaluated using GIS (Geographic Information System), RS (Remote Sensing), and other technologies [34].

Although in recent years ecological restoration has become a hot topic, research on ecological restoration lacks systematic induction and sorting, and the overall progress of research needs to be further explored. At the same time, given the large number of studies in this field, relevant methods, such as bibliometrics, are required to quickly understand the research progress. Bibliometrics can help to understand trends in the published literature in a given research field, the journals and disciplines involved, the authors, countries, and institutions, as well as the collaboration between them [35]. In addition, the main topics and future research directions of a specific field can be understood from the keywords and topics of the literature [36]. Bibliometrics research promotes a field in a novel and meaningful way to lay a solid foundation. It enables scientists to master a wealth of information about their research field, to derive new research ideas, and to locate their expected future contributions to the field [7,37,38].

In this study, to determine the development of research in the field of ecological restoration and gain a deeper understanding of the future trend, an R runtime environment was set up, and the Bibliometrix series were used to systematically summarize and sort publications in the field of ecological restoration published in the Web of Science database between 1990 and 2022. The research objectives are as follows:

1. To provide an overview of the research and major research forces in the field of ecological restoration from 1990 to 2022 (countries/regions, institutions, publications, journals, among others).
2. To analyze the popular research topics in this field and their characteristics.
3. To explore potential research directions based on emerging trend analysis.

## 2. Materials and Methods

### 2.1. Method

Bibliometric analysis can help determine publication patterns and objectively evaluates the research status quo and development process for different countries, regions, scientific research institutions, or authors in specific fields using quantitative research methods such as mathematical statistics. Bibliometrics methods excel at exploring the underlying knowledge structures contained in the academic literature and at integrating visualized results to further analyze the field. Bibliometrics software can be used to quantitatively analyze large amounts of literature data and generate visualization and content analysis results. The visualization approach allows for a clearer relationship between the various research scopes and a scientifically effective understanding of the development direction and trends of scientific research [39,40]. Bibliometrix, developed by Massimo Aria and Corrado Cuccurullo in 2017, is a currently widely used R language software package for bibliometric analysis and scientific visualization [41]. The scientific knowledge map generated by the software package reflects the development trend of a certain discipline or knowledge field in a certain period and facilitates an accurate understanding of the evolution of a given scientific frontier. Content analysis effectively combines qualitative and quantitative analysis. It merges the relevant contents of the research object and statistical data to finally draw a qualitative conclusion [42]. Literature metrology, visualization, and content analysis methods are combined to analyze the literature related to the research field, which can objectively evaluate and explore the research status and development trend of a certain field.

Here, we used the Bibliometrix software package to quantify parameters such as the number of publications, countries, institutions, journals, and citations. We employed Excel, the R language, and other software packages to draw charts. Content analysis and visualization include the analysis of highly cited literature and of cooperative networks of research institutions, as well as clustering analysis of high-frequency keywords. All this can be performed using the Bibliometrix package.

### 2.2. Data and Processing

Web of Science is the world’s largest and most comprehensive academic information resource, covering more than 12,000 academic journals in a wide range of disciplines, including natural sciences, engineering, biomedical sciences, social sciences, arts, and humanities [43]. In this paper, the Web of Science core database was taken as the data retrieval source, the search scope was selected to be “subject”, and the search term TS = (“Ecological rehabilitation” OR “Ecological remediation” OR “Ecological restoration”) was applied. The time span of the search was 1990 to August 2022, and the literature type was limited to “Article”, “Review”, and “Database Review”. The retrieval language was set to English. Overall, 23,755 documents in the field of ecological restoration were obtained after data deduplication, irrelevant data removal, and other preprocessing steps. The retrieved data were saved as plain texts.

After data collection, the articles were analyzed using the Bibliometrix software package. First, we analyzed the evolution of the volume and citations of the documents in the field of ecological restoration for the period from 1990 to 2022. Second, we analyzed the main research disciplines involved in ecological restoration as well as the countries and institutions with a larger number of published articles. Third, we identified the major journals in the field of ecological restoration, along with their JCR (Journal Citation Reports) divisions, If (Impact Factor) and the volume of published literature in the field of ecological restoration between 1990 and 2022. Finally, we conducted a visual analysis of keywords and themes in the field to identify hot research topics and trends.

**Figure 1:**
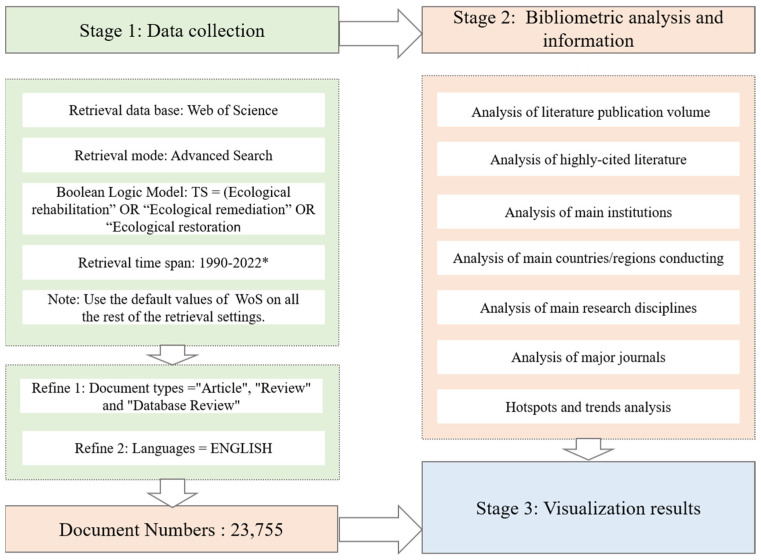
Bibliometrics process in the field of ecological restoration. (* as of 31 August 2022).

Figure 1 shows a summary of the bibliometrics process in the field of ecological restoration.

## 3. Results and Analysis

### 3.1. Analysis of the Publication Volume in the Field of Ecological Restoration

**Figure 2:**
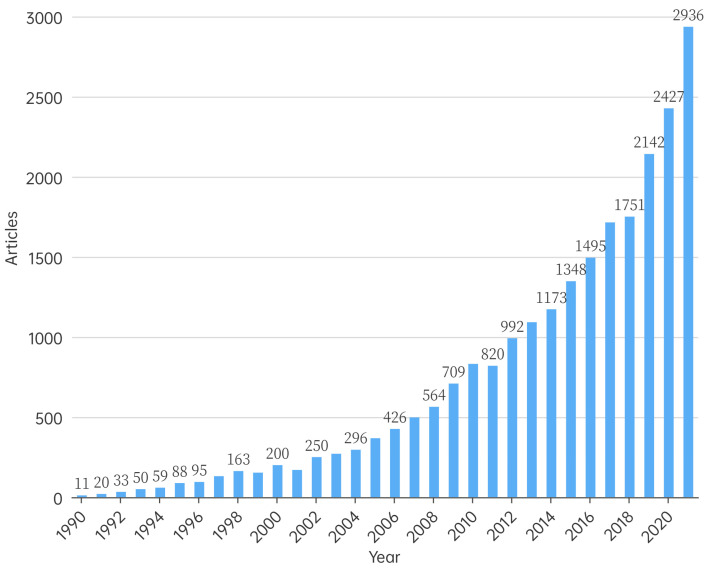
Volume of published articles on ecological restoration from 1990 to 2022.

Based on the statistics of the number of published articles in each year, the research process of each field can be understood to a certain extent. From 1990 to 2021, the number of publications in the field of ecological restoration increased gradually (Figure 2), indicating that ecological restoration has attracted increased attention. From 1990 to 1996, the number of published articles was less than 100 per year, and the total number of published literatures was 356, accounting for only 1.50% of the total articles. The annual increase in the number of articles was slow, and this period can be termed the “initial stage”; publications mainly focused on qualitative analysis. Subsequently, from 1997 to 2017, the number of published articles increased considerably, with the annual number of published articles increasing from 131 to 1715, indicating that during this period, ecological restoration research was gaining more importance. As more scientists published in this field, issues such as ecological damage, ecological restoration theories, measures, technologies, policies, and other aspects became more prominent, with considerable interdisciplinarity. After 2017, the number of ecological restoration publications continued to increase substantially, reaching 2936 in 2021.

### 3.2. Analysis of Literature Citations in the Field of Ecological Restoration

#### 3.2.1. Annual Citation Trend Analysis

**Figure 3:**
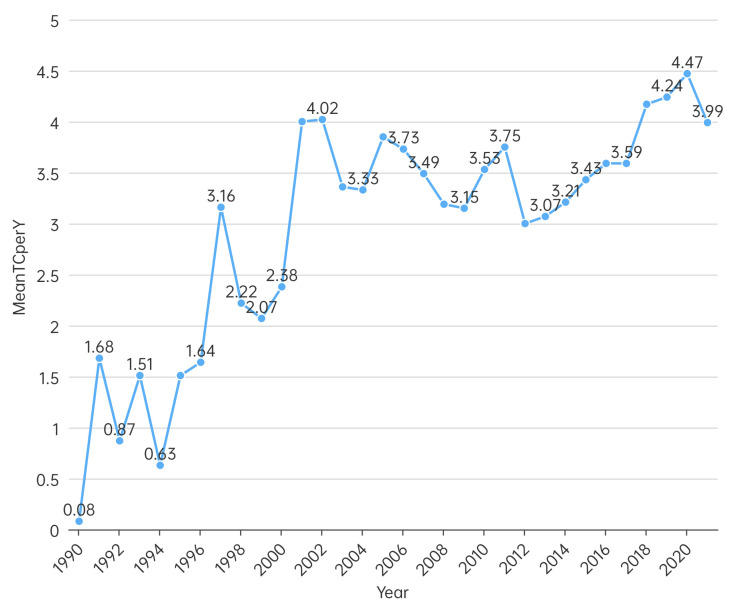
Average annual citations in the field of restoration ecology from 1990 to 2021.

The average annual citation frequency of the literature in the ecological restoration field showed a fluctuating increase (Figure 3). The citation frequency was low from 1990 to 1996; even in 1996, the year with the highest citation frequency, it was as low as 1.64, indicating that this research field was still in its infancy and did not receive widespread attention. The average citation frequency during 1997–2002 and 2003–2022 was 2.97 and 3.58, respectively, with a peak in 2020 (4.47). To some extent, the citation frequency is closely related to the development stage of the research. In general, the increasing number of articles over time indicates the growing influence of the field of ecological restoration. 

#### 3.2.2. Analysis of Highly Cited Articles

Ecological restoration has received considerable interest from relevant institutions and researchers, with most of the important findings published in high-ranking journals (Table 1). Among the studies on ecological restoration published from 1990 to 2022, the most frequently cited globally was “Historical overfishing and the recent collapse of coastal ecosystems” by Professor Jeremy B. Jackson [44] of the University of California, San Diego, which was published in *Science* in 2001; this publication received 4346 citations worldwide. In their article, the authors suggest that more specific palaeoecological, archaeological, and historical data should be obtained to provide a framework for the restoration of coastal ecosystems by considering the extensive human disturbances to coastal ecosystems from a historical perspective and to construct achievable goals for the restoration and management of coastal ecosystems. It provides a novel perspective, namely a historical perspective, and laid the foundation for coastal ecosystem restoration. This has triggered a wave of research on driving factors such as pollution, water degradation, and climate change [45,46], as well as measures to restore coastal ecosystems [47,48]. The second most frequently cited article was “The value of estuarine and coastal ecosystem services” by Professor Edward B. Barbier [49] of the University of Wyoming, which was published in *Ecological Monographs* in 2011, with 2626 citations worldwide. This study assesses the value of estuarine and coastal ecosystems such as swamps, mangroves, nearshore coral reefs, seagrass beds, sandy beaches, and dunes and factors driving their decline. The authors propose the protection and enhancement of the immediate and long-term values of estuarine and coastal ecosystems by improving regulatory, institutional, and legal frameworks and developing measures to rehabilitate them. This article laid the foundation for the restoration of estuarine and coastal ecosystems and triggered an upsurge in research on the factors affecting estuarine and coastal ecosystems [50], mangrove restoration [51], and swamp restoration [52]. The third most frequently cited article was “Mycorrhizal fungal diversity determines plant biodiversity, ecosystem variability and productivity” by Professor Marcel G. A. van der Heijden [53] of the University of Basel, published in *Nature* in 1998, with 2301 citations worldwide. This study shows that microbial interactions can drive ecosystem functions such as plant biodiversity, productivity, and variability and postulates the protection of microorganisms to ensure the successful management and restoration of various ecosystems. Microbial restoration is suggested to restore various degraded ecosystems. Other scientists picked up this issue and performed comprehensive studies in the fields of soil salinization [54], wetland restoration [55], and grassland restoration [56], among others. 

### 3.3. Main Countries/Regions Conducting Research in the Field of Ecological Restoration

**Figure 4:**
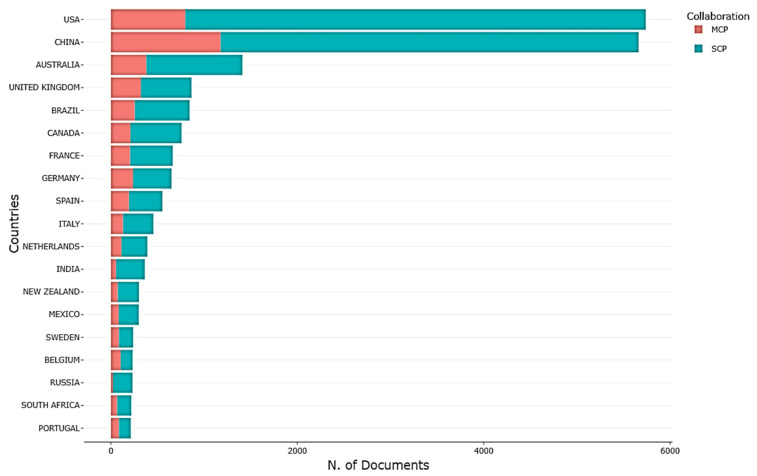
Top 10 countries in terms of literature volume in the field of ecological restoration. MCP = Multiple-country publications; SCP = Single-country publications.

We identified the top ten countries conducting research in this field based on the literature volume (Table 2, Figure 4). Although many countries around the world performed studies in restoration ecology, the ten countries with most of the publications were the USA, China, Australia, the UK, Brazil, Canada, France, Germany, Spain, and Italy. Of these, the USA, China, Australia, the UK, and Brazil each published over 843 studies, with the USA and China publishing 5738 and 5662 studies, respectively. The publications of the top five countries respectively accounted for 19.10%, 12.66%, 8.58%, 6.60%, and 5.47% of the total publications and collectively for over 45% of the total publications. This indicates that these countries contributed the most to research on ecological restoration. For example, in the US, studies on the dynamic changes and mechanisms of ecosystems after destruction and disturbance were conducted, investigating northern broadleaf and mixed forests, among others. Australian scientists mainly investigated the degradation of arid land and the restoration of coniferous forests in the cold temperate zone [57].

In addition, the numbers of single-country populations (SCP) and multiple-country populations (MCP) can be used to further analyze the scientific research strength of a given country. Throughout the study period, China had the highest MCP at 1176, whereas the USA had the highest SCP at 4940. This indicates that US scientists largely performed independent research, whilst their Chinese counterparts were more engaged in international collaboration. As ecological restoration research received increasing attention from scientists, regionalization and globalization became the focus of ecological restoration research.

### 3.4. Main Institutions Conducting Research in the Field of Ecological Restoration

To obtain an understanding of the research strength of individual institutions, we determined the top ten research institutions based on their publication volume (Table 3), which were as follows: University of the Chinese Academy of Sciences, Chinese Academy of Sciences, Beijing Normal University, American Forest Service, Arizona State University, Northwestern University, University of Western Australia, University of Queensland, University of Sao Paulo, and Institute of Geographic Sciences and Resources Research. The University of the Chinese Academy of Sciences published 602 articles in this specific field, followed by the Chinese Academy of Sciences with 566 articles and Beijing Normal University with 561 articles. As the top three institutions were Chinese ones, China attaches great importance to ecological restoration research. In recent years, with the introduction of the Master Plan of National Major Projects for the Protection and Restoration of Important Ecosystems (2021–2035), China has committed itself to the systematic protection, overall restoration, and comprehensive management of mountains, rivers, forests, fields, lakes, and grasslands. Institutions in the US, Australia, and Canada have followed this example.

**Figure 5:**
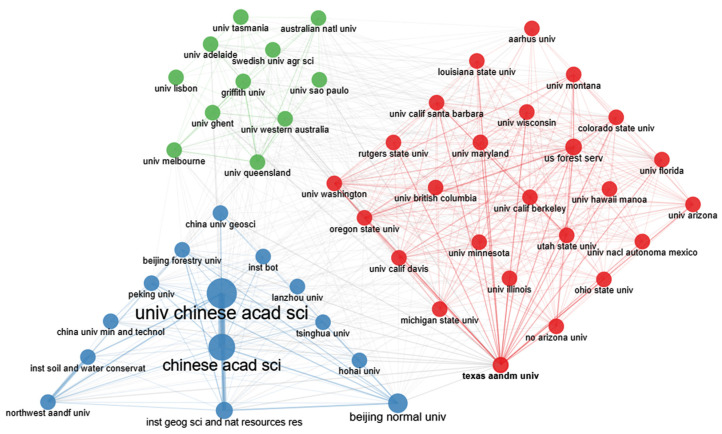
Collaboration network of institutions publishing articles in the field of ecological restoration from 1990 to 2022.

We also mapped a collaboration network among 50 institutions (Figure 5), using institutions as units of analysis to showcase published papers and collaborations. The radius size of the circles in the figure is proportional to the number of studies published collaboratively by the institutions, and the thickness of the line between two circles indicates the collaboration intensity. The thicker the line, the more intense the collaboration between institutions and vice versa. Clearly, institutions from China, the USA, and Australia ranked highly in terms of the number of articles published in collaboration with other institutions. Collaborations were mostly found between institutions of the same country, whereas large-scale multinational institutional collaboration groups have not yet been established. Regarding global ecological restoration research, there is a need to strengthen the cooperation between institutions and deepen research from multiple perspectives in the future.

### 3.5. Main Research Disciplines in the Field of Ecological Restoration

**Figure 6:**
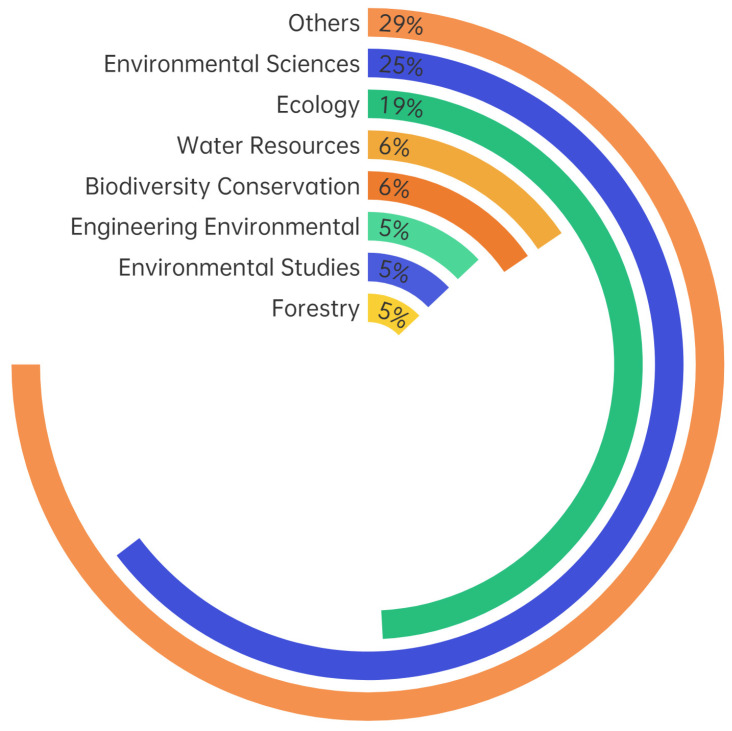
Average annual citations in the field of ecological restoration from 1990 to 2021.

Bibliometric analysis was conducted on the disciplines covered by the ecological restoration research in the Web of Science to explore the major disciplines; the results are shown as percentages. Generally, the field of ecological restoration presented a trend of interdisciplinary development (Figure 6). Among the many research disciplines, environmental science was the dominant one, accounting for 25% of the total, followed by ecology (19%), biodiversity conservation (6%), water resources (6%), forestry (5%), environmental studies (5%), and engineering environment (7%). Other disciplines, such as marine and freshwater biology, multidisciplinary geosciences, plant science, and soil science, did not account for more than 5% of the total. The key issues of ecological restoration research in environmental science involved environmental monitoring as well as water, air, and soil pollution control. The discipline of ecology is closely related to that of biodiversity conservation. Ecological restoration research in the discipline of ecology focuses on the adaptation and feedback effects of ecosystem processes to global changes, ecosystem service functions, and biodiversity conservation. In the discipline of biodiversity conservation, ecological restoration studies aim to protect endangered plants and animals and the various biological resources on Earth.

### 3.6. Main Journals Publishing Articles in the Field of Ecological Restoration

This section shows the most prolific journals in the field of ecological restoration and analyzes their leading indicators (Table 4). The top ten journals were published in the Netherlands, Switzerland, Germany, the UK, and the USA, covering a variety of fields such as environmental science and ecology, engineering technology, and agriculture and forestry sciences. The most productive journals were *Ecological Engineering*, *Ecological Indicators*, and *Science of the Total Environment*. *Ecological Engineering*, which was divided into the Q2 area by JCR partition in 2021, with an impact factor of 4.379, published 502 articles on ecological restoration from 1990 to 2022. *Ecological Indicators*, which was divided into the Q2 area by JCR partition in 2021, with an impact factor of 6.363, published 392 articles on ecological restoration from 1990 to 2022. *Science of the Total Environment*, which was divided into the Q2 area by JCR partition in 2021, with an impact factor of 10.753, published 311 articles on ecological restoration from 1990 to 2022.

The subject areas of these three journals are closely related to the field of ecological restoration research. Specific topics covered in *Ecological Engineering* include habitat reconstruction, ecotechnology, synthetic ecology, bioengineering, restoration ecology, ecology conservation, ecosystem rehabilitation, stream and river restoration, reclamation ecology, and non-renewable resource conservation. Specific topics covered by *Ecological Indicators* include broader assessment objectives and methods, e.g., biodiversity, biological integrity, and sustainability, through the use of indicators, resource-specific indicators such as landscape, agroecosystems, forests ecosystems, aquatic ecosystems, and wetlands. Specific topics covered by *Science of the Total Environment* include environmental remediation of soil and groundwater, nanomaterials, microplastics, and other emerging contaminants, novel contaminant (bio)monitoring and risk assessment approaches, stress ecology in marine, freshwater, and terrestrial ecosystems, trace metals and organics in biogeochemical cycles, water quality, and security.

### 3.7. Hot Research Topics and Trends in the Field of Ecological Restoration

#### 3.7.1. High-Frequency Keyword Analysis

Keywords highly generalize the content of a research article [58]. High-frequency keyword analysis well reflected the current hot issues in the field of ecological restoration research. In bibliometrics, keywords are considered to represent the foundational elements of the knowledge concepts and are widely used to reveal the knowledge structure of the field of study.

**Figure 7:**
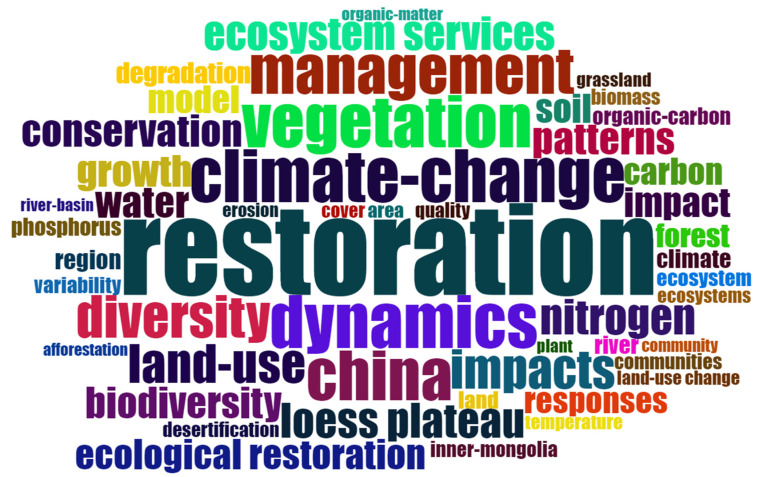
Cloud graph of high-frequency keywords in the field of ecological restoration from 1990 to 2022.

The top 50 keywords were plotted in a keyword cloud graph (Figure 7), with larger fonts indicating a higher frequency of occurrence. High-frequency keywords included “Management”, “Conservation”, “Biodiversity”, “Vegetation”, “Climate-change”, “Ecosystem services”, “Patterns”, and “Land use”. We noted that there was a wide range of studies in the field of ecological restoration, which is considered a solution to ecosystems issues and is closely related to climate change, land use, and management strategies, among others. In global ecological restoration research, biodiversity, ecosystem services, climate change, land use, ecological restoration patterns, and ecosystem management and protection, among other factors, were focused on. Among them, enhancing biodiversity and ecosystem services enables ecosystem restoration and regeneration [59]. Vulnerable ecosystems that have been damaged by climatic changes and human activities are closely linked to ecological restoration. Different restoration patterns are applicable to ecosystems of different types (such as soils, forests, or lakes) and the science of the restoration pattern determines the effectiveness of ecological restoration. Ecological patterns are established based on the theory of bioremediation, focusing on applying ecological principles to degraded ecosystems, combined with one or more of the corresponding ecological techniques for restoration [60,61]. In addition, the management and protection of the ecosystem by the government is crucial and will largely determine the success of ecological restoration programs.

Specifically, the field of ecological restoration research can be divided into numerous areas, such as forest, river, soil, and plant restoration, according to the restoration objectives. With respect to the restoration scale, large-scale ecological restoration (such as on the Loess Plateau region of China), meso-scale ecological restoration (such as that of rivers, forests, or wetlands), and small-scale (such as that of mines and landfill sites) ecological restoration projects can be distinguished. Among the specific issues, soil erosion, desertification, grassland degradation, forest resource shortages, water shortages, biodiversity reduction, and climate change can be considered [62]. At the same time, ecological restoration research needs to be combined with socio-economic aspects, such as ecological migration [63] or fishing bans [64]. In addition, the evaluation of ecological restoration effects is an important issue [65].

#### 3.7.2. Cluster Analysis and Multiple Correspondence Analysis of High-Frequency Keywords

**Figure 8:**
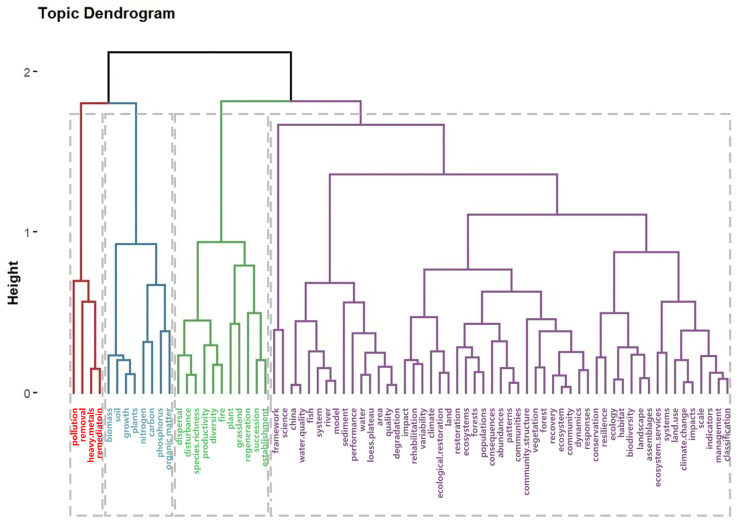
Dendrogram of the system cluster analysis of keywords in the field of ecological restoration from 1990 to 2022.

Cluster analysis is a common method in bibliometrics, and in statistics, it is a multivariate statistical analysis method for studying the “clustering of things” [66,67]. In this study, hierarchical clustering was applied to first take the keyword of each cluster as a category; subsequently, the keywords were merged into a higher-level cluster based on similarity, and finally, all individuals were grouped into categories. Here, we obtained four categories (Figure 8).

The first category is mainly related to “Heavy metals”, “Pollution”, “Removal”, and “Restoration”. It focuses on exploring and removing heavy metals from contaminated soil, river ecosystems, and other ecosystems. The heavy metal industry is an important economic pillar for the development of a country’s regional economy, providing raw materials for industrial production. However, years of continuous mineral extraction have led to large areas of wasteland and severe damage to ecosystems. Heavy metals are characterized by high toxicity and low solubility. Wastewater from heavy metal extraction, smelting, and casting in industrial production is often discharged directly into the soil and surface water and has gradually become a key factor threatening ecosystem health [68,69]. The second category is mainly related to “Biomass”, “Soil”, “Nitrogen”, and “Carbon”. Studies in this category focus on soil microbial biomass carbon and nitrogen concentrations. Soil microorganisms play an important role in terrestrial ecosystem restoration as major decomposers. Carbon and nitrogen are often considered the most important elements in terrestrial ecosystems as their interactions play a key role in the global biogeochemical cycle and ecosystem functions [70,71]. The third category is mainly related to “Species richness”, “Diversity”, and “Grassland”, with a focus on grassland ecological restoration. Due to overgrazing, large-scale development, and construction, grassland resources have been seriously damaged. Grassland landscape fragmentation has been intensified, the ecological functions have declined, and the normal operation rules of the food chain, energy flow, and material circulation of grassland ecosystems have been seriously disturbed [72]. As generally, a higher species richness means a faster ecosystem restoration, there is an urgent need to increase grassland species richness to restore grassland resources [73]. The fourth category is mainly related to “Climate change”, “Water quality”, “Land use”, “Ecosystem services”, “Model”, and “Framework”, with an emphasis on frameworks and the evaluation of the effects of ecological restoration models. Ecological restoration effect evaluation mainly involves water quality, climate change, land use change, and ecosystem services [74,75,76].

#### 3.7.3. Thematic Evolution Analysis

**Figure 9:**
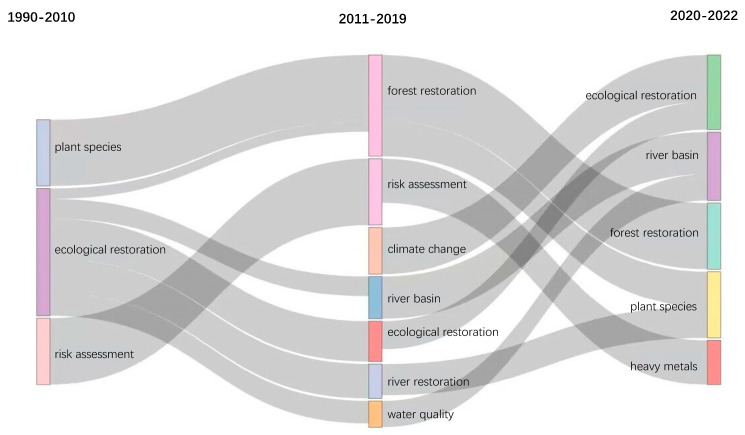
Evolution of research topics in the field of ecological restoration from 1990 to 2022.

Research topic and anticipated research direction evolve over time. The research direction of ecological restoration has undergone tremendous changes in the past 32 years. In Figure 9, the rectangles and squares from left to right depict the chronological order of the topic evolution. The topic development from 1990 to 2010 is shown on the left, that from 2011 to 2019 is shown in the middle, and that from 2020 to 2022 is shown on the right. The keywords are connected by grey lines of different shapes that connect rectangles of various colors. Based on the temporal distribution of the key topics, we summarized the development trend of ecological restoration research.

Before 2011, ecological restoration research was in its infancy, and scientists started to explore subjects such as “Risk assessment” and “Plant species”. From 2011 to 2019, studies on ecological restoration were gradually enriched, with topics such as “Forest restoration”, “Risk assessment”, “Climate change”, “River basin”, and “Water quality”, presenting a trend of multidisciplinary and multi-perspective comprehensive analysis. In 2019, the United Nations announced the implementation of the Decade for Ecosystem Restoration (2021–2030) initiative to address biodiversity loss, climate damage, and increased pollution. From 2020 to 2022, ecological restoration research mainly focused on “Forest restoration”, “Plant species”, “River basin”, and “Heavy metals”. Forests represent the largest carbon pools, which makes them important in climate change mitigation. Generally, for every 1 m^3^ stock volume growth of forests, an average of 1.83 t of carbon dioxide is absorbed, and 1.62 t of oxygen is released. Forest restoration will therefore contribute to the worldwide achievement of the ‘carbon neutrality’ target by 2060 [77,78]. Some governments and international organizations have launched forest restoration programs. For example, the German government and the International Society for the Conservation of Nature launched the “Bonn Challenge”, which aims to restore 150 million hectares of forest by 2020 [79]. The New York Declaration On Forests aims to restore 350 million hectares of degraded or logged land to forests by 2030 [80]. With the rapid development of the social economy, science and technology, industry and construction, and heavy metal pollution seriously threatens the safety of soil and river ecosystems. The number of studies on the technologies and methods for treating heavy-metal-polluted sites is constantly increasing, with theoretical and practical significance [81]. River basins are the basic units of hydrological response and the spatial scale for studying ecological restoration. The mechanisms of interaction between landscape patterns and hydrological processes at this scale are complex [82]. River basin restoration includes, but is not limited to, dam removal/renovation, fishway construction, flow modification, floodplain reconstruction, stormwater management, natural shoreline protection, habitat protection and restoration, riverbank stabilization, vegetation restoration, river channel reconstruction, water quality management, and ecological regulation [83,84].

## 4. Discussion

Ecosystems not only provide a variety of raw materials or products that are directly used by humans but also have various functions such as carbon storage, climate regulation, pollution purification, water conservation, soil and water conservation, wind prevention and sand fixation, and biodiversity maintenance [85]. In this sense, ecosystems guarantee the sustainable development of human society and the economy. With the rapid development of society and the economy, ecosystems are increasingly disturbed and destroyed by human activities [86]. In response to ecosystem degradation, biodiversity loss, and climate change caused by human activities, the concept and practice of ecological conservation and restoration are rapidly developing across the globe. Many countries/regions, such as China, India, and New Zealand, have set short-term and long-term development goals for ecological restoration [87,88,89]. Such detailed goals and tasks oscillate around one or several aspects of ecological restoration to address the many ecological issues than need to be solved. At the same time, academic research on ecological restoration has increased significantly over the past three decades, with significant positive trends.

The results show that the number of articles in the field of ecological restoration has been increasing continuously since 1990. Before 1996, few articles were published, and the research progress was slow. However, after 1996, ecological restoration received increased attention, and the development of this research field was accelerated. Various theories have been proposed, such as the community succession, the ecosystem stability, the community construction, and the ecological niche theory. Of these, the community succession theory has been widely used in the restoration of degraded forest, grassland, and wetland vegetation [90]. In addition, studies also focused on restoration techniques and measures for different objects. For example, grassland restoration techniques include no-till reseeding, livestock reduction, grassland cultivation, grassland tillage, conversion of farmland to grassland, and fertilization [91]. River restoration technologies mainly include physical restoration, such as aeration and aeration; sediment dredging; chemical remediation, such as the introduction of chemicals that react with pollutants; and bioremediation, namely the use of aquatic animals, plants, and microorganisms to absorb, degrade, and transform pollutants [92]. 

However, the research perspectives are not limited to the direct study of ecological restoration, and climate change, land use, policy making, and other related aspects are also considered. For example, man-made grassland degradation caused by overgrazing, climate change, and other driving factors is one of the most serious global disturbances, affecting about 49% of the global grassland area, with serious impacts on livelihoods, biodiversity, and ecosystem functions [93]. The rate of forest extinction has been accelerated by the rapid decline of forest resources due to the expansion of arable land and excessive commercial logging for fuel and infrastructure materials [94]. The study of ecological restoration policies is also crucial to the management and protection of ecosystems and largely determines the success of ecological restoration. The co-occurrence of keywords and the evolution of this theme also show that the four hotspots in this field are heavy metal pollution remediation, soil microbial biomass carbon and nitrogen concentrations, grassland ecological restoration, and ecological restoration effect evaluation frameworks and models. The main research directions include watershed restoration, heavy metal removal, and forest restoration.

In the future, cooperation between countries/regions should be strengthened to carry out ecological restoration research from multiple perspectives. European and American countries are leading the way in ecological restoration, not only because they have taken the lead in relevant research but also because of the extensive and close collaboration between countries. At present, there is still a large gap between the ecological restoration statuses of developing countries and those of developed countries. Therefore, it is of great significance to strengthen the cooperation with other countries to promote the healthy development of the ecological system [95]. Most ecological restoration studies only focus on the protection and restoration of specific ecosystems and targets such as river basins, heavy metal pollution, and forests. In the future, the research scope of ecological restoration should also be transformed from local and single-ecosystem ecological restoration to large-scale and global ecological restoration involving multiple ecosystems, with the aim to initiate multi-object systematic and multi-scale ecological restoration research. 

From the study of the protection and restoration of specific ecosystems and targets, research has gradually shifted to the mechanism and path of the successful realization of ecological restoration. At the same time, ecological restoration is not only a natural and ecological process but also a social and economic process, involving economic loss, economic input, value restoration, and resource development and management, with a series of human value orientation motivations and goals. The integration of the conceptual framework and research methods of natural science and humanities in ecological restoration projects is gaining increasing attention [96]. Therefore, ecological restoration is not only a scientific but also a social and economic issue. In the future, an important direction of ecological restoration research is to cross-study ecological restoration with social disciplines and economic disciplines, such as supporting mechanisms of ecological restoration, including policy, legal, and management mechanisms [97,98]. In addition, the evaluation of ecological restoration effects is crucial and should involve water quality, climate change, land use change, and ecosystem services in the fields of ecology, environmental science, engineering, and geography, among others. 

Multidisciplinary research in the field of ecological restoration is still in its infancy [99]. Integrating multidisciplinary theories to formulate ecological restoration policies and research frameworks for adaptation and deepening the system of multidisciplinary theories and methods will help optimize the evaluation system of ecological restoration effects and make ecological restoration more scientific and comprehensive [100]. In the future, it might be important to establish a scientific, reasonable, authoritative, and effective evaluation system according to local conditions.

The results obtained so far are encouraging. However, studies on ecological restoration are scattered and ignore the systematic characteristics of the research objects, which makes it difficult to form a unified logical main line and connect different research perspectives. In this paper, we look at the development of ecological restoration research from a macro perspective, determining the major research journals, institutions, countries, and disciplines and analyzing the findings of the major articles. With this approach, we clarify the cognitive context of the academic circle, help scientists to understand the hotspots and directions of ecological restoration, and provide references for in-depth research on ecological restoration. Although this paper has a certain contribution to the research field, it also has some shortcomings. For example, only the keywords “Ecological rehabilitation”, “Ecological remediation”, and “Ecological restoration” were selected. Other keywords, such as “Land reclamation” and “Comprehensive land management”, are also related to the field of ecological restoration, but they were not considered. The retrieval period was 1990 to 2022, although certainly, some important results were already published before 1990. Our study is based on a single database, the Web of Science core database, and ignores articles from Scopus, CNKI, and other databases. Therefore, in the following research, we will explore how to merge other databases and expand our research scope based on the current discussion. Finally, ecological restoration research involves many aspects and issues. In the future, the development trend of important branch issues will be further analyzed.

## 5. Conclusions

In this paper, we looked at the development of ecological restoration research from a macro perspective, identifying the trends in publications, major research journals, institutions, countries, and articles. The evolution of the research hotspots and themes was analyzed and the future research directions were discussed. From 1990 to 2022, the number of articles published in the field of ecological restoration research increased gradually, and the fluctuation of the annual average citation number increased. The most important articles were published in high-ranking journals. The US was the leading research country in this field, followed by China and Australia. American scientists seemed to prefer independent research, whereas Chinese scholars engaged more in international collaboration. The top three institutions in this field were located in China, with UNIV CHINESE ACAD SCI being the most prolific one. The field shows the interdisciplinary development trend of environmental science, ecology, and biodiversity conservation. The top three prolific journals were *Ecological Engineering, Ecological Indicators*, and *Science of the Total Environment.*

The results also indicated that the research field of ecological restoration covers a wide range, with biodiversity, ecosystem services, climate change, land use, and ecological restoration theories and technologies being the main topics. The four hotspots were heavy metal pollution, soil microbial biomass carbon and nitrogen concentrations, grassland ecological restoration, and evaluation frameworks and modeling of ecological restoration’s effects. At present, river basin restoration, heavy metal removal, and forest restoration are the main research directions in the field of ecological restoration. In the future, multi-object systematic research and multi-scale ecological restoration research should be strengthened, along with improvement of the ecological restoration effect evaluation system, and ecological restoration research must incorporate social and economic issues. We try to clarify the cognitive context of the academic circle, help scientists to understand the hotspots and directions of ecological restoration, and provide references for in-depth research on ecological restoration.

## Figures and Tables

**Table 1:** Top 10 most cited publications in the field of ecological restoration from 1990 to 2022.

Rank	Paper	DOI	Year	Journal	TC
1	JACKSON JBC, 2001, SCIENCE	10.1126/science.1059199	2001	*Science*	4346
2	BARBIER EB, 2011, ECOL MONOGR	10.1890/10-1510.1	2011	*Ecological Monographs*	2626
3	VAN DER HEIJDEN MGA, 1998, NATURE	10.1038/23932	1998	*Nature*	2301
4	BELLWOOD DR, 2004, NATURE	10.1038/nature02691	2004	*Nature*	2128
5	BUNN SE, 2002, ENVIRON MANAGE	10.1007/s00267-002-2737-0	2002	*Environmental Management Volume*	2112
6	ORTH RJ, 2006, BIOSCIENCE	10.1641/0006-3568	2006	*BioScience*	1944
7	MYERS RA, 2003, NATURE	10.1038/nature01610	2003	*Nature*	1927
8	WALSH CJ, 2005, J N AM BENTHOL SOC	10.1899/04-020.1	2005	*Journal of the North American Benthological Society*	1903
9	WALSH CJ, 2005, J N AM BENTHOL SOC-a	10.1899/04-020.1	2005	*Journal of the North American Benthological Society*	1903
10	DE GROOT RS, 2010, ECOL COMPLEX	10.1016/j.ecocom.2009.10.006	2010	*Ecological Complexity*	1862

Note: TC = total citation number.

**Table 2:** Top 10 countries in terms of the literature volume in the field of ecological restoration.

Rank	Country	Number of Articles	Single-Country Publications (SCP)	Multiple-Country Publications (MCP)
1	USA	5738	4940	798
2	China	5662	4486	1176
3	Australia	1410	1030	380
4	United Kingdom	864	543	321
5	Brazil	843	587	256
6	Canada	758	549	209
7	French	662	456	206
8	Germany	650	413	237
9	Spain	552	358	194
10	Italy	456	326	130

**Table 3:** Top 10 research institutions in terms of literature volume in the field of ecological restoration.

Rank	Research Institution	Country	Number of Articles Published
1	University of Chinese Academy of Sciences	China	602
2	Chinese Academy of Sciences	China	566
3	Beijing Normal University	China	561
4	American Forest Service	USA	452
5	Arizona State University	USA	415
6	Northwestern University	USA	390
7	University of Western Australia	Australia	381
8	University of Queensland	Australia	358
9	University of Sao Paulo	Canada	339
10	Institute of Geographic Sciences and Resources Research	China	329

**Table 4:** Top 10 journals in terms of literature volume in the field of ecological restoration from 1990 to 2022.

Rank	Journal	Country	Journal Citation Reports (JCR)	Impact Factor (If)	Articles
1	*Ecological Engineering*	Netherlands	Q2	4.739	502
2	*Ecological Indicators*	Netherlands	Q2	6.363	392
3	*Science of the Total Environment*	Netherlands	Q2	10.753	311
4	*Sustainability*	Switzerland	Q2	3.889	188
5	*Remote Sensing*	Switzerland	Q1	5.349	151
6	*Water*	Switzerland	Q2	3.530	133
7	*Environmental Science and Pollution Research*	Germany	Q2	5.190	131
8	*International Journal of Environmental Research and Public Health*	Switzerland	Q1	4.614	117
9	*Land Degradation and Development*	England	Q2	4.377	115
10	*Journal of Cleaner Production*	USA	Q1	11.087	107

## Data Availability

All relevant datasets in this study are described in the manuscript.
